# Constitutional trisomy 8 mosaicism as a model for epigenetic studies of aneuploidy

**DOI:** 10.1186/1756-8935-6-18

**Published:** 2013-07-01

**Authors:** Josef Davidsson, Srinivas Veerla, Bertil Johansson

**Affiliations:** 1 Division of Clinical Genetics, Department of Laboratory Medicine, Lund University, SE 221 85, Lund, Sweden; 2 Department of Oncology/SCIBLU DNA Microarray Resource Centre, Lund University, SE 221 85, Lund, Sweden; 3 Department of Clinical Genetics, University and Regional Laboratories, Region Skåne SE 221 85, Lund, Sweden

**Keywords:** Trisomy, Methylation, Gene Expression

## Abstract

**Background:**

To investigate epigenetic patterns associated with aneuploidy we used constitutional trisomy 8 mosaicism (CT8M) as a model, enabling analyses of single cell clones, harboring either trisomy or disomy 8, from the same patient; this circumvents any bias introduced by using cells from unrelated, healthy individuals as controls. We profiled gene and miRNA expression as well as genome-wide and promoter specific DNA methylation and hydroxymethylation patterns in trisomic and disomic fibroblasts, using microarrays and methylated DNA immunoprecipitation.

**Results:**

Trisomy 8-positive fibroblasts displayed a characteristic expression and methylation phenotype distinct from disomic fibroblasts, with the majority (65%) of chromosome 8 genes in the trisomic cells being overexpressed. However, 69% of all deregulated genes and non-coding RNAs were not located on this chromosome. Pathway analysis of the deregulated genes revealed that cancer, genetic disorder, and hematopoiesis were top ranked. The trisomy 8-positive cells displayed depletion of 5-hydroxymethylcytosine and global hypomethylation of gene-poor regions on chromosome 8, thus partly mimicking the inactivated X chromosome in females.

**Conclusions:**

Trisomy 8 affects genes situated also on other chromosomes which, in cooperation with the observed chromosome 8 gene dosage effect, has an impact on the clinical features of CT8M, as demonstrated by the pathway analysis revealing key features that might explain the increased incidence of hematologic malignancies in CT8M patients. Furthermore, we hypothesize that the general depletion of hydroxymethylation and global hypomethylation of chromosome 8 may be unrelated to gene expression regulation, instead being associated with a general mechanism of chromatin processing and compartmentalization of additional chromosomes.

## Background

Constitutional trisomy 8 mosaicism (CT8M) is a relatively rare chromosomal disorder with an estimated frequency of approximately 1/25,000 to 1/50,000 [1]. However, since the phenotypes of individuals with CT8M vary quite extensively, ranging from severe malformations with impaired cognitive functioning to rather discrete dysmorphic changes [2], the true prevalence may well be higher. Characteristic clinical features of CT8M include elongated facial features, abnormally shaped ears, strabismus, camptodactyly, clinodactyly, deep plantar and palmar skin furrows, vertebral/hip anomalies, cardiovascular and urogenital malformations and mild to moderate mental retardation [3]. As regards acquired trisomy 8, it is one of the most common abnormalities in malignant myeloid disorders, such as acute myeloid leukemia, myelodysplastic syndromes and myeloproliferative neoplasms [4,5]. Interestingly, based on several case reports on myeloid malignancies in patients with CT8M [see Additional file 1: Table S1], CT8M seems to be associated with an increased risk of these disorders. It has even been suggested, albeit in a small patient series, that the ‘acquired’ +8 in myeloid diseases in some instances could represent an unrecognized CT8M [6]. This, however, remains to be confirmed or refuted in larger studies.

There is ample evidence that CT8M arises post-zygotically through a mitotic error, with no preferential parental origin of the gained chromosome 8 (chr8) [7-11]. The pathogenetically important consequence of CT8M is most likely, as for numerical abnormalities in general, a dosage effect, with overexpression of genes on the gained chromosome [12]. However, it is unknown whether all chr8 genes are upregulated in CT8M. In a previous study [10], a single gene (*GSR* at 8p) was investigated in two patients and shown to have an increased activity. To date, no global gene expression analyses of CT8M cases have been reported, but considering that myeloid malignancies with acquired trisomy 8 overexpress most, but not all, genes on this chromosome [13-15], it is plausible that the same holds true also for CT8M. Furthermore, it is also unclear whether CT8M is associated with epigenetic changes involving chr8, akin to the methylation changes on chr21 in Down syndrome (DS) [16,17]. We have previously shown that most tri- and tetrasomic chromosomes in high hyperdiploid (51 to 67 chromosomes) acute lymphoblastic leukemia (ALL), one of the most common subtypes of childhood ALL [18], are globally less methylated than their disomic counterparts and suggested that this could be a general phenomenon associated with gains of chromosomes, irrespective of whether they are constitutional or acquired [19]. However, this has, until the present study, not been investigated.

In order to further our understanding of gene expression patterns and epigenetic mechanisms associated with gain of chr8, we used CT8M as a model, allowing analyses of single cell-derived fibroblast clones, harboring either trisomy or disomy 8, from the same patient. This model enables comparisons between identical cell types (for example, fibroblasts), differing only in respect to chr8 content, from a single individual and, hence, circumvents the bias introduced when comparing the epigenetic findings with unrelated, healthy individuals. We used this model to profile global gene and microRNA (miRNA) expression as well as genome-wide and gene promoter specific DNA methylation and hydroxymethylation patterns in trisomic and disomic fibroblasts from a patient with CT8M, using microarrays and methylated DNA immunoprecipitation (MeDIP).

## Results

### Unsupervised HCA of gene and miRNA expression

A standard unsupervised hierarchical cluster analysis (HCA), after using a variance filter with a cut-off value of 50% of the highest standard deviating genes to limit the number of probes and a Pearson correlation test [20] for average linkage clustering, was used to investigate similarities and differences in global gene and miRNA expression patterns among disomy 8, trisomy 8, and references. Unsupervised HCA of only gene expression and of both gene and miRNA expression data clustered the trisomy 8 cultures together but did not differentiate between disomy 8 and reference cultures [see Additional file 2: Figure S1], whereas unsupervised HCA of only miRNA expression data was less accurate in clustering the various culture groups [see Additional file 2: Figure S1]. To test for a possible bias introduced by *in vitro* culturing, we performed long-term culturing (16 weeks) of disomy 8 and trisomy 8 cells and repeated the analysis. Unsupervised HCA clustered the long-term cultures together with their short-term counterparts, indicating a minimal impact of *in vitro* effects on gene and miRNA expression [see Additional file 2: Figure S1]. It should be stressed that we cannot exclude the possibility of an effect of culturing as such during the first eight weeks compared with uncultured cells. However, since analyses could not be performed without expansion of single cell clones, this could not be addressed in the present study.

### PCA and supervised HCA of gene and miRNA expression

**Figure 1:**
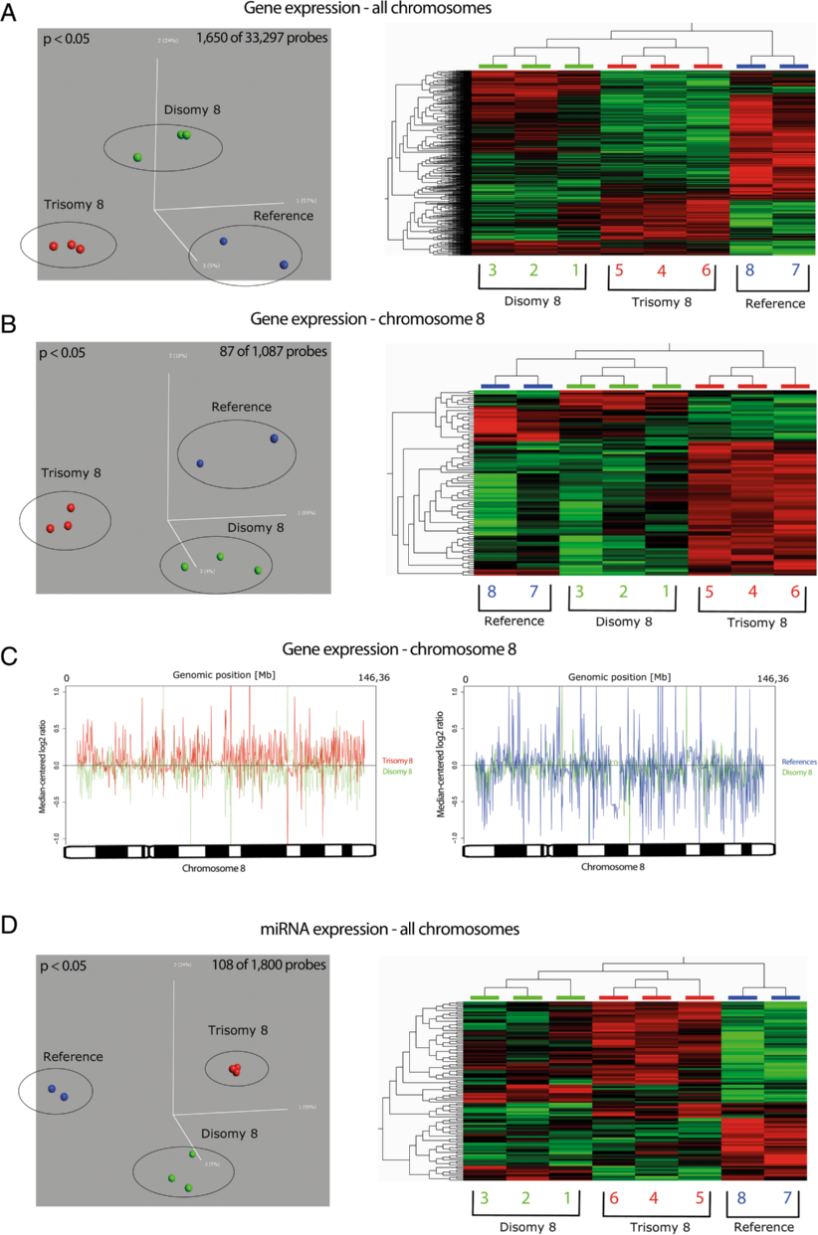
**PCA and supervised HCA of gene and miRNA expression accurately cluster the trisomy 8, disomy 8, and reference cultures. (A)** The PCA identified 1,650 probes that displayed significant expression differences among the trisomy 8, disomy 8, and reference groups (left). Supervised HCA of these probes accurately clustered the subgroups, as illustrated in the heat map to the right. **(B)** A similar pattern was observed when analyzing only chr8 data, with the PCA identifying 87 genes (left) and HCA accurately clustering the subgroups (right), placing the trisomy 8 cultures in a single branch. **(C)** Median-centered gene expression of chr8 demonstrated that the majority of the genes on this chromosome in the trisomy 8 cultures were upregulated compared with the disomy 8 cultures (left), whereas few differences were observed when comparing the disomy 8 cultures with the reference cultures (right). **(D)** The PCA identified 108 miRNA probes that displayed significant expression differences between the trisomy 8, disomy 8, and reference groups (left). Supervised HCA of these probes grouped the cultures correctly (right). chr8, chromosome 8; HCA, hierarchical cluster analysis; miRNA, microRNA; PCA, principal component analysis.

Principal component analysis (PCA) identified 1,650 unique probes (5% of all 33,297 probes analyzed in our expression dataset) that displayed significant expression differences among the trisomy 8, disomy 8, and reference groups. Supervised HCA of these probes correctly clustered the three groups, with the trisomy 8 cultures being placed in the same branch as the disomy 8 cultures (Figure 1A). When performing PCA only of chr8 probes (n = 1,087), 87 significant genes (12% of the 728 known HG19 protein-coding genes on this chromosome) were identified. Supervised HCA of these genes again accurately clustered the three groups, this time with the trisomy 8 cultures being placed in a single branch (Figure 1B).

To illustrate the expression levels of genes on chr8, the average median-centered gene expression in the trisomy 8, disomy 8, and reference cultures was plotted against the location of each gene on this chromosome, revealing a global overexpression of chr8 genes in the trisomy 8 cultures compared with both the disomy 8 and reference cultures (Figure 1C and Additional file 3: Figure S2); in contrast, the expression levels in the disomy 8 and the reference cultures were very similar (Figure 1C). In total, among the 728 protein-coding genes on chr8, 476 (65%) were overexpressed in the trisomy 8 cells compared with the disomy 8 cells, as ascertained by measuring the median expression values in the two culture groups.

PCA and supervised HCA of global miRNA expression, based on 108 significant probes, also correctly clustered the three groups, placing the trisomy 8 cultures in the same branch as the disomy 8 cultures (Figure 1D). Analysis of only chr8 miRNA expression was uninformative because of too few differentially expressed probes (data not shown).

### Genes and miRNAs possibly contributing to the CT8M phenotype and their clinical and biological functions

A multiple testing approach identified 25 protein-coding genes, one small nucleolar RNA, and one miRNA that were significantly overexpressed and four genes and one miRNA that were significantly underexpressed in the trisomy 8 cultures as compared with both the disomy 8 and reference cultures. The over- or underexpression of seven of the genes identified was validated by real-time quantitative PCR (qPCR) [see Additional file 4: Figure S3]. As seen in Table 1, 10 (31%) of these 32 genes/miRNAs are located on chr8. In addition, a *t*-test of the global gene expression patterns in the trisomy 8 versus the disomy 8 cultures revealed 502 differentially expressed genes. The top clinical conditions and biological features associated with these genes, as identified using an Ingenuity pathway analysis (Ingenuity Systems, Redwood City, CA, USA), are provided in Additional file 5: Table S2.

**Table 1:** Genes and miRNAs significantly associated with the presence of trisomy 8

**Gene/RNA symbols**	**Chr position**^**a**^	**Function/process/component**^**a**^	**Fold change**^**b**^
*DES*	2q35	Cytoskeleton	11.8762965
*CHSY3*	5q23.3	Transferase	2.6232108
*EDNRA*^c^	4q31.22	G-protein coupled receptor activity	2.55418755
*CRYAB*^c^	11q23.1	Small heat shock protein	2.3484328
*KCTD20*	6p21.31	Ion transport	2.33678525
*SCRN1*	7p14.3	Exocytosis	2.09512935
*POPDC2*	3q13.33	Membrane associated protein	2.0460034
*LDB3*	10q23.2	Cytoskeleton	2.0369906
*ZNF185*	Xq28	Protein-protein interactions	2.03330695
*LRP12*^c^	8q22.3	Lipoprotein receptor related protein	1.936707
*AGPAT6*^c^	8p11.21	Transferase	1.72648325
*ENTPD4*^c^	8p21.2-21.3	Hydrolase	1.7125851
*RAB3GAP2*	1q41	Exocytosis	1.6605436
*COX6C*	8q22.2	Respiratory electron transport chain	1.63403665
*TNFRSF10A*	8p21.3	Apoptosis	1.6329978
*MIR151*	8q24.3	Post-transcriptional regulation	1.6319659
*FNBP1*	9q34.11	Protein binding	1.59805445
*TNKS*	8p23.1	Transferase	1.5799036
*RAB2A*	8q12.1	GTPase	1.5733374
*SLC4A3*	2q35	Ion transport	1.47450405
*ELP3*	8p21.1	Regulation of transcription	1.4660848
*OSTM1*	6q21	Osteoclast differentiation	1.4356524
*DMPK*	19q13.32	Kinase	1.4022396
*PFN2*	3q25.1	Cytoskeleton	1.3931477
*SNORD115-32*	15q11.2	Small nucleolar RNA	1.37446365
*RPS6KA1*	1p36.11	Kinase	1.34011185
*INTS10*	8p21.3	Small nuclear RNA processing	1.3153658
*CASP4*^d^	11q22.3	Apoptosis	−1.45109678
*ARRDC4*	15q26.2-26.3	Signal transduction	−1.498037415
*KITLG*	12q21.32	Signal transduction	−1.66295902
*CPXM2*^d^	10q26.13	Peptidase	−1.785911
*MIR424*	Xq26.3	Post-transcriptional regulation	−3.3616748

^a^The chromosome (Chr) positions are based on GRCh37 in Ensembl (http://www.ensembl.org), whereas the functions/processes/components are based on NCBI Gene (http://www.ncbi.nlm.nih.gov/gene); ^b^compared to the baseline expression in the dataset; ^c^overexpression confirmed by quantitative real-time PCR [see Additional file 4: Figure S3].

^d^underexpression confirmed by quantitative real-time PCR [see Additional file 4: Figure S3]. miRNAs, microRNAs; NCBI, National Center for Biotechnology Information.

### Hypermethylated and hyperhydroxymethylated promoters/CpG islands in relation to gene expression

An edge-preserving smoother analysis identified 150 to 200 genes that were highly enriched for 5-methylcytosine (5 mC) and/or 5-hydroxymethylcytosine (5 hmC) in the disomy 8, trisomy 8, and reference groups, respectively. As seen in Additional file 6: Table S3, approximately 80% of the hypermethylated genes were underexpressed, whereas only a minority of the hyperhydroxymethylated genes was under- or overexpressed, as ascertained by a *t*-test. When analyzing only genes on chr8, a similar pattern emerged – 17 (89%) of 19 hypermethylated genes were underexpressed, whereas only 8 (32%) of 25 hyperhydroxymethylated genes were underexpressed.

### The patterns of hypermethylated and hyperhydroxymethylated promoters/CpG islands

**Figure 2:**
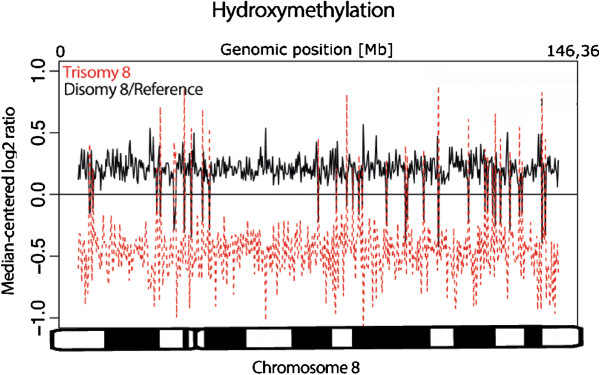
**Promoter-specific hydroxymethylation levels on chromosome 8 are significantly lower in the trisomy 8 compared with the disomy 8/reference cultures.** The levels of average promoter-specific hydroxymethylation on chr8 in the trisomy 8 and in the disomy 8 and reference cultures combined were log2-converted, median-centered, and plotted against genomic positions. Significantly lower levels (*P <*0.05; *t*-test) were observed in the trisomy 8 cultures. chr8, chromosome 8.

**Figure 3:**
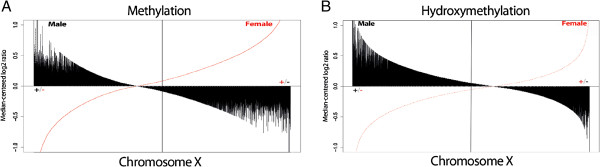
**Promoter-specific methylation and hydroxymethylation on the X chromosome in the female culture is higher and lower, respectively, compared with the male cultures.** A rank plot of median-centered promoter-specific methylation and hydroxymethylation log2 levels on the X chromosome reveals that the female reference culture generally displays higher levels (*P* <0.05; *t*-test) of methylation compared with **(A)** the average levels in the male cultures and **(B)** lower levels (*P* <0.05; *t*-test) of hydroxymethylation compared with the male cultures.

The levels of promoter-specific methylation on the autosomes in the trisomy 8 group did not differ significantly from the corresponding levels in the disomy 8 and reference groups combined [see Additional file 7: Figure S4], nor did the levels of 5 hmC, with the notable exception of chr8 on which the 5 hmC levels were significantly lower (*P* <0.05; *t*-test) in the trisomy 8 group (Figure 2 and Additional file 8: Figure S5). As regards the X chromosome, its promoters/CpG islands were generally (irrespective of gender and culture group) significantly (*P* <0.05; *t*-test) less hydroxymethylated than all ‘autosomal’ promoters/CpG islands [see Additional file 9: Figure S6]. The female reference displayed a higher level of promoter-specific methylation on the X chromosome (Figure 3A) as well as a lower level of promoter-specific hydroxymethylation (Figure 3B) compared with the XY cultures.

### Genome-wide methylation patterns

**Figure 4:**
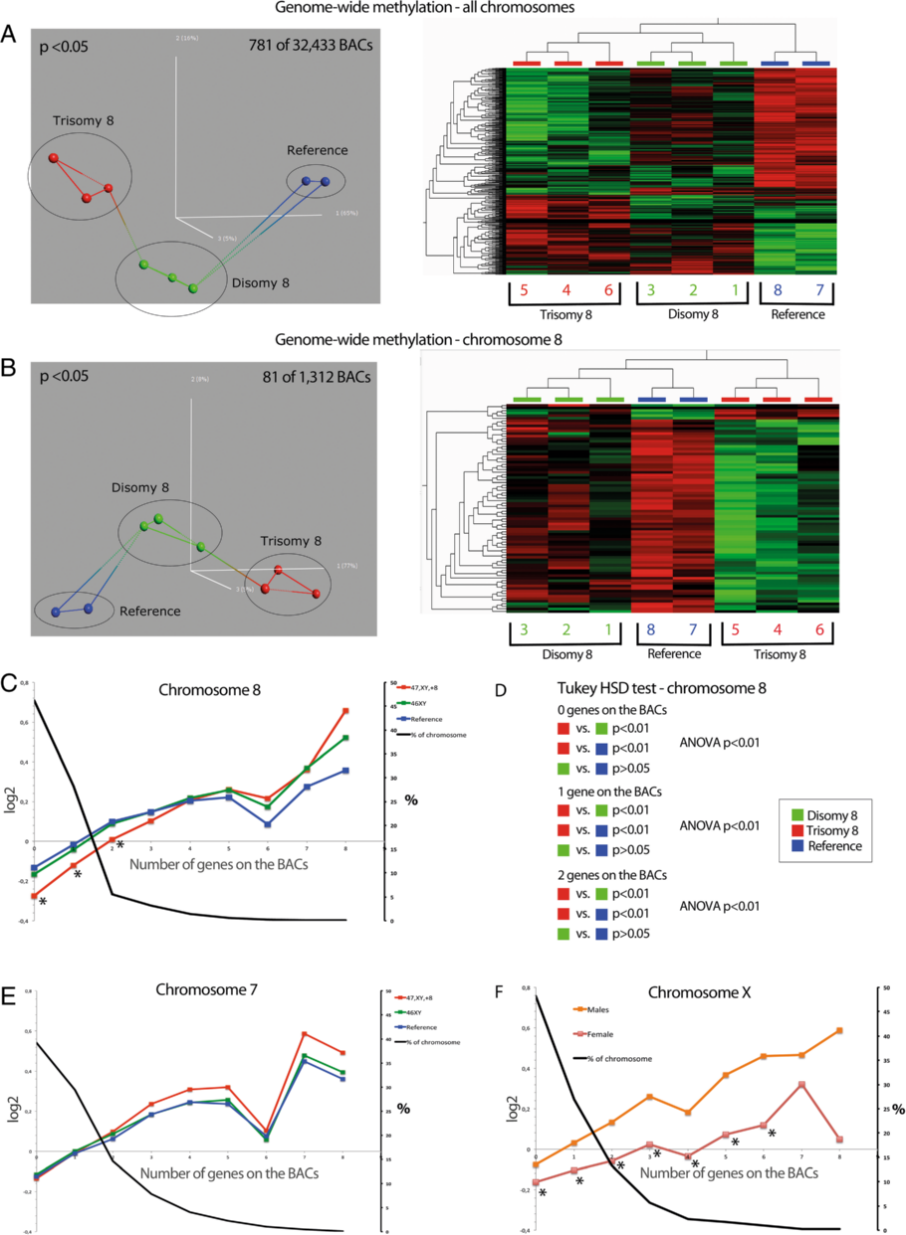
**Accurate clustering of the trisomy 8, disomy 8, and reference cultures by PCA and supervised HCA of genome-wide methylation patterns and significant hypomethylation of gene-poor regions on chromosome 8 only in the trisomy 8 cultures. (A)** The PCA identified 781 BACs that displayed significant differences between the trisomy 8, disomy 8, and reference cultures (left). Supervised HCA of these BACs accurately clustered the subgroups, as illustrated in the heat map (right). **(B)** A similar pattern was observed when analyzing only chr8 data, with the PCA identifying 81 BACs (left) and HCA accurately clustering the subgroups (right). **(C)** Mean log2 ratio of the three culture subgroups plotted against the number of genes on the BACs for chr8 revealed a significant hypomethylation of gene-poor regions in trisomy 8 cultures. The black curve shows the proportions that the BAC clones with different number of genes constitute of all BAC clones on chr 8; for example, the BACs with no genes comprise 45% of all the BAC clones on this chromosome. **(D)** An independent and weighted ANOVA and a Tukey HSD test verified the global hypomethylation of gene-poor regions on chr8 in trisomy 8 cultures but not in the disomy 8 or reference cultures. **(E)** Mean log2 ratio of the three culture subgroups plotted against the number of genes on the BACs for chromosome 7 (used as an example of another autosome) revealed no significant differences in genome-wide methylation. **(F)** Mean log2 ratio of female and male cultures plotted against the number of genes on the BACs for chromosome X revealed significant hypomethylation of gene-poor regions only in the female culture. ANOVA, analysis of variance; BAC, bacterial artificial chromosome; chr8, chromosome 8; HCA, hierarchial cluster analysis; HSD, honestly significant difference; PCS, principal component analysis.

PCA identified 781 unique clones, comprising 2.4% of the 32,433 bacterial artificial chromosomes (BACs) in the array, that displayed significant (*P* <0.05) differences in methylation between the trisomy 8, disomy 8, and reference groups. Supervised HCA of these clones clustered the three groups, with the trisomy 8 cultures being placed in the same branch as the disomy 8 cultures (Figure 4A). PCA of only BACs on chr8 revealed 81 significant clones (6.2% of the 1,312 BACs covering chr8), and supervised HCA of these BACs again accurately clustered the three groups, this time with the trisomy 8 cultures constituting a single branch (Figure 4B).

An independent and weighted ANOVA and a Tukey honestly significant difference (HSD) test identified a significant (*P* <0.01) global hypomethylation of gene-poor regions on chr8 in trisomy 8 cultures but not in the disomy 8 or reference cultures (Figure 4C and D). This pattern was not observed on any of the other autosomes (Figure 4E). However, a similar global hypomethylation of gene-poor regions was seen on the X chromosome in the female reference compared with all XY cultures, irrespective of sample type (Figure 4F).

## Discussion

Aneuploidy is a well-studied phenomenon in human cancer as well as in various model organisms, for example *Arabidopsis thaliana*, *Drosophila melanogaster*, *Mus musculus*, and *Saccharomyces cerevisiae*, that in recent years has received increased attention as regards its effects on cellular physiology, transcriptional consequences, and genomic instability [21-24]. Such studies have revealed that aneuploidy is associated with a general proliferative disadvantage, species-conserved stress related gene expression patterns regardless of gained chromosome as well as increased and defect mitotic recombination and DNA repair, resulting in genomic instability. In humans, constitutional aneuploidy, for example, loss or gain of sex chromosomes or trisomy of chromosomes 13, 18, or 21, is a major cause of miscarriage and developmental disturbances. Prior to the present study, gene expression patterns in patients with constitutional trisomies have exclusively been compared with expression signatures derived from disomic control cells from unrelated healthy individuals [25-31]. The differences previously observed might, hence, not only represent the presence of a specific trisomy but also interindividual variations and comparisons between non-identical cell types, with the latter introducing severe interpretation difficulties. For this reason, we used single cell cloning of fibroblasts from an individual with CT8M to ascertain, in an unbiased manner, the epigenetic changes associated with gain of chr8. Because several human chromosomal disorders occasionally, or even frequently, occur as postzygotic events, resulting in two or more chromosomally different cell lines developed from a single zygote, the present approach is applicable also for epigenetic studies of other constitutional mosaicisms, with well-known examples including, apart from CT8M, triplications of 8p, 9p, 12p, and 18p, trisomies for chromosomes 9, 13, 14, 16, 18, 20, 21, and 22, and Turner and Klinefelter syndromes [32].

By single cell cloning with subsequent gene expression and methylation analyses, we here show that trisomy 8-positive fibroblasts from a patient with CT8M display a characteristic expression and methylation phenotype that is clearly distinct from both disomy 8 cells and normal reference fibroblasts. In fact, PCA and HCA of global as well as chr8-specific gene/miRNA expression and methylation profiles grouped all the trisomy 8 cultures in the same cluster branch, with no discernible effect by long-term culturing [see Additional file 2: Figure S1]. When investigating which genes were the major ‘expression contributors to the observed clustering, we could, not surprisingly, demonstrate that the majority of the genes on chr8 in the trisomy 8 cultures were overexpressed compared with the chr8 genes in the disomy 8/reference groups (Figure 1C and Additional file 3: Figure S2 and Additional file 4: Figure S3). Hence, it seems safe to assume that the clinical features as well as the elevated risk for malignant myeloid disorders associated with CT8M [3,4] [see Additional file 1: Table S1] is, at least partly, due to a direct chr8 gene dosage effect. However, far from all deregulated genes and non-coding RNAs in the trisomy 8-positive cells were located on chr8 (Table 1), nor were all genes on this chromosome up-regulated (Figure 1C). Thus, all genes on chr8 are not dosage sensitive and trisomy 8 directly or indirectly affects genes situated on other chromosomes as well. That not every gene on a constitutional trisomic chromosome displays increased expression has previously been clearly demonstrated in DS; in fact, only a minority of the chr21 genes are overexpressed in DS [25-28].

Pathway analysis of the deregulated genes in the trisomy 8 cells revealed that ‘cancer’ and ‘genetic disorder’ were the top two biological functions in the diseases and disorders category and that ‘hematological system development and function’ and ‘hematopoiesis’ were top ranked in the physiological system development and function category [see Additional file 5: Table S2]. The latter may explain the increased incidence of myeloid neoplasms in CT8M patients [4]. Furthermore, by using a set of stringent inclusion criteria, several candidate genes (Table 1) for some of the clinical manifestations were identified [see Additional file 10: Table S4]. For example, the presence of congenital heart disease could possibly be ascribed to the deregulation of the *DES*, *LDB3*, *POPDC2* and *SLC4A3* genes [see Additional file 11: Figure S7], all of which have previously been associated with cardiac function and pathology, such as cardiomyopathy. In addition, eight of the candidate genes (*CASP4*, *COX6C*, *FNBP1*, *LRP12*, *RAB2A*, *RPS6KA1*, *TNFRSF10A* and *TNKS*; Table 1) have been associated with various solid tumors. These genes also displayed a joint functional association [see Additional file 11: Figure S7]. The overexpression of *MIR151*, which was observed in the trisomy 8 cultures only, is noteworthy because this non-coding RNA has been suggested to play a role in metastases [see Additional file 10: Table S4]. Furthermore, the deregulation of *AGPAT6*, *CPXM2*, *CRYAB, EDNRA*, *ENTPD4*, *RAB2A* and *RAB3GAP2* as well as their functional network [see Additional file 11: Figure S7] may contribute to the mild to moderate mental retardation seen in CT8M patients since these genes have all previously been associated with neurological development and function. In addition, *ELP3* and *SNORD115-32*, involved in small nucleolar RNA processing and transcript elongation, were upregulated in the trisomy 8 cells. They could also play a role in the cognitive impairment, considering that ELP3 regulates the maturation of projection neurons and that SNORD115-32 has been implicated in splicing defects of the serotonin receptor 2C in Prader-Willi syndrome [see Additional file 10: Table S4].

The present analyses of global methylation and hydroxymethylation patterns in trisomy 8-positive cells may provide insights into the epigenetic consequences of aneuploidy. As a proof-of-principle, we initially investigated methylation and hydroxymethylation of the X chromosome in XX and XY cultures (Figure 3). As expected, unlike its active counterpart, the inactivated X chromosome (Xi) in the XX cells exhibited hypomethylation of gene-poor regions but hypermethylation of a substantial proportion of promoters (Figures 3A and 4F); the latter is considered a sex chromosome dosage compensation mechanism [33-35]. Furthermore, and in agreement with a previous study [36], we observed a general depletion of 5 hmC on the X chromosome compared with all autosomes in both males and females [see Additional file 9: Figure S6]. Interestingly, similar methylation and hydroxymethylation profiles of chr8 were observed in the trisomy 8 cells, which – compared with the disomic cells – displayed depletion of 5 hmC (Figure 2) and a global hypomethylation of gene-poor regions (Figure 4C). However, unlike the Xi, no elevated levels of promoter/CpG island 5 mC content on chr8 were detected [see Additional file 7: Figure S4]. It is interesting to note that the methylation status of chr8 observed in the present study is reminiscent of the hypomethylation patterns of acquired chromosomal gains reported in neoplasia, that is, hypomethylation of gene-poor regions of the tri-/tetrasomic chromosomes in high hyperdiploid childhood ALL and of trisomies 7 and 14 in colon cancer [19,37]. Thus, we suggest that an association between aneuploidy and globally lowered levels of 5 mC on the gained chromosomes is valid for both constitutional and acquired numerical changes. It should be emphasized, however, that the hypomethylation seems to be confined to genomic regions with no or few genes and this phenomenon may, hence, be unrelated to gene expression changes. Instead, it is possible that the hypomethylation of gene-poor regions may be involved in chromatin compartmentalization. In fact, it has been demonstrated that absence of methylation influences chromatin organization by increasing the nuclear clustering of pericentric heterochromatin [38]. It is hence feasible that the hypomethylation of gene-poor regions results in altered nuclear positioning of trisomic/tetrasomic chromosomes. However, such an effect on the nuclear architecture remains to be addressed experimentally.

The biological impact of the decreased hydroxymethylation levels of the gene promoters/CpG islands on chr8 in the trisomy 8 cells and on the X chromosome is currently unclear. In fact, it is debated whether the main cellular role of 5 hmC is to be a demethylation intermediate or if it constitutes a bona fide epigenetic regulator, attracting chromatin and transcriptional modifiers by itself. To date, no specific 5 hmC-binding protein has been found, arguing against the latter mechanism [39]. Whether a demethylation intermediate or not, it is clear that 5 hmC is situated primarily in the gene-bodies of actively transcribed genes [40], playing an important role in cellular lineage commitment as well as in tissue-specific gene expression [41,42]. In support of this, it has recently been demonstrated [43] that the most important predictor of 5 hmC content in a cell is the tissue type from which it is derived and that the levels of hydroxymethylation tend to decrease rapidly over time when cells are cultured *in vitro*. This possible bias was circumvented in our study by the use of identical cell types as well as culture times. Interestingly, global loss of 5 hmC has been associated with a variety of human malignancies [44,45] with an inverse relationship between 5 hmC and cell proliferation [46]. Hence, the hypohydroxymethylation on chr8 in the trisomy 8-positive cells may contribute to the propensity for myeloid malignancies in CT8M patients.

## Conclusions

In this study we used a mosaic genetic syndrome, namely constitutional trisomy 8 mosaicism, to investigate the epigenetic mechanisms associated with aneuploidy using the patient’s own diploid cells as control. This new approach circumvents the interpretation difficulties introduced when comparing trisomic cells with diploid cells from an unrelated control. We profiled gene and miRNA expression as well as DNA methylation and hydroxymethylation patterns. The trisomic fibroblasts displayed a characteristic expression and methylation patterns, with the majority of chromosome 8 genes in the trisomic cells being overexpressed. However, not all deregulated genes were located on this chromosome. Thus, trisomy 8 affects genes situated also on other chromosomes, which in cooperation with the gene dosage effect might influence the clinical features and elevated cancer risk seen in this syndrome. Interestingly, trisomy 8-positive cells also displayed a general depletion of hydroxymethylation and a global hypomethylation of gene-poor regions on chromosome 8, thus partly mimicking the patterns seen in the inactivated X-chromosome. This novel finding could be associated with a general mechanism of chromatin processing of additional chromosomes.

## Methods

### Patient history and cytogenetic analyses

In 2002, a cytogenetic analysis of peripheral blood from a 10-year-old boy with a history of developmental delay, cryptorchidism and pectus excavatum revealed trisomy 8 in three of 25 analyzed metaphases. To confirm the diagnosis of CT8M, a skin biopsy was analyzed in 2003, revealing the karyotype 47,XY,+8[5]/46,XY[20].

### Single-cell cloning of fibroblasts from the patient with CT8M

The primary culture for cytogenetic analysis of fibroblasts was established using standard methods in our department, including initial mechanical disaggregation and subsequent culture in RPM1 1640 (Invitrogen, Carlsbad, CA, USA). After the chromosome analysis, the fibroblasts were viably frozen in liquid nitrogen. For the present study, the primary culture was thawed and cultured in standard conditions with RPMI 1640 medium (Invitrogen) containing 10% FBS (Sigma-Aldrich, St. Louis, MO, USA) in a 50 ml (25 cm^2^) cell culture flask (BD Falcon, Stockholm, Sweden). The culture was then, at the time of subconfluence, passaged 1:3 by treatment with 0.5% trypsin. One subculture was refrozen in liquid nitrogen, one was analyzed by interphase fluorescence in situ hybridization (FISH) using CEP-8 (Abbott Molecular, Des Plaines, IL, USA) to evaluate the proportions of cells with either trisomy or disomy 8, and one was used for further passages. After a short culture time of the latter, the cells were trypsinized, counted in a Bürker chamber (Digital Bio, Seoul, Korea), and diluted so that approximately one cell could be plated per well in 96-well microtiter plates (BD Falcon). Viable confluent cell cultures were used for further passages at a ratio of 1:1 to 24-well microtiter plates (BD Falcon). After reaching confluence, all cell cultures were transferred 1:1 to 6-well microtiter plates (BD Falcon). The cell cultures were then resuspended, after which a fraction from each was used for interphase FISH analysis with dual (Cy3 and Cy5) CEP-8 probes (Abbott Molecular). A minimum of 285 (95%) of 300 normal or abnormal nuclei was used as a cut-off to designate a culture as being either 46,XY or 47,XY,+8. Three cultures with disomy 8 and three with trisomy 8 were then passaged to 250 ml (75 cm^2^) cell culture flasks (BD Falcon). Finally, these six cell cultures were harvested simultaneously, and RNA and DNA were extracted. The total time in culture was eight weeks. Additional file 12: Figure S8 shows a schematic overview of the culture process. The regional ethics board at Lund University approved the study and informed consent was obtained from the patient’s guardians in accordance with the Declaration of Helsinki.

### Reference fibroblasts

Primary dermal fibroblasts from a male neonate and a female adult were obtained from the American Type Culture Collection (Manassas, VA, USA). The cells were maintained and passaged in RPMI 1640 medium (Invitrogen) containing 10% FBS (Sigma-Aldrich) in 250 ml cell culture flasks (BD Falcon). The cultures were cytogenetically characterized using conventional G-banding analysis, revealing normal male and female karyotypes, respectively.

### Global gene and miRNA expression analysis

The mirVana kit (Invitrogen) was used to isolate RNA, including miRNA, from the fibroblast cultures from the CT8M patient and the references. The RNA concentration was measured with a NanoDrop 1000 spectrophotometer (Saveen & Werner AB, Malmö, Sweden), and the quality of the RNA was assessed with a 2100 Bioanalyzer (Agilent Technologies, Santa Clara, CA, USA). Total RNA was then hybridized to the Affymetrix Human Gene 1.0 ST and the GeneChip miRNA arrays (Affymetrix, Santa Clara, CA, USA) according to the manufacturer’s protocols. Quality control and initial analyses were performed using the Expression Console and the miRNA QC Tool (Affymetrix).

### Bioinformatic analyses of gene and miRNA expression

HCA, PCA, multiclass significance analysis of microarrays (SAM), and *t*-tests were used to compare expression profiles between fibroblasts with either disomy 8 (from the CT8M patient and from the references) or trisomy 8 (from the CT8M patient) and to identify differentially expressed genes and miRNAs in the trisomic cells. Filtering, HCA, and PCA of log2 transformed expression data were performed using the Qlucore Omics Explorer v 2.0 (Qlucore, Lund, Sweden). For PCA, based on the Pearson correlation matrix, the data were normalized by setting the mean to 0 and σ to 1 and by filtering the variance on the basis of σ/σ_max_. The Benjamini-Hochberg method was applied for error correction by q-value calculation. A *P*-value of <0.05 was used as a cut-off. HCA of the genes identified by PCA was performed on the basis of Euclidean metric distance (samples) and Pearson correlation average linkage test (genes).

Differentially expressed genes and miRNAs were identified using the correlation matrix-based PCA in combination with SAM and *t*-tests (MultiExperiment Viewer v 4.2) [20]. A multiple testing approach was applied, with the criteria for inclusion being set to a *P*-value of <0.05 for the *t*-tests and a *q*-value of ≤10% in the SAM analysis, when comparing trisomy 8 with disomy 8 (CT8M only) and when comparing trisomy 8 with disomy 8 (CT8M and references grouped together). Only genes and miRNAs significantly differentially expressed in all the above-mentioned statistical analyses were considered to be associated with CT8M. Functional association networks of these genes were identified by the GeneMANIA analysis (http://www.genemania.org/) [47].

### qPCR validation

The qPCR analyses were performed using standard protocols for predesigned TaqMan probes (Applied Biosystems, Carlsbad, CA, USA) to validate over- or underexpression of genes significantly associated with the presence of trisomy 8 in the global gene expression analyses (Table 1). The expression of transcripts of the following seven genes was ascertained: *AGPAT6* (Hs00410940_m1), *CASP4* (Hs01031951_m1), *CRYAB* (Hs00157107_m1), *CPXM2* (Hs00406866_m1), *EDNRA* (Hs03988672_m1), *ENTPD4* (Hs01555223_m1), *LRP12* (Hs00940649_m). Fold expression was calculated relative to endogenous control normalization of *TBP* (Hs.590872_m1), encoding a TATA box binding protein. The rationale for using *TBP* as control was its uniform expression in all cultures in the global gene expression analyses. All qPCR analyses were performed in triplicate and assayed on a 7500 real-time PCR system (Applied Biosystems) and ΔΔCT values were calculated using the comparative CT method [48].

### MeDIP

From each sample (fibroblasts from the CT8M patient and the references), 12 μg of DNA was diluted in standard *Tris-ethylenediaminetetraacetic acid (*TE) buffer to a concentration of 75 ng/μl. The DNA was sonicated for 90 seconds with a Covaris S220 (Covaris, Woburn, MA, USA) set to a 5% duty cycle, intensity value 3, and 200 cycles per burst to generate fragments ranging from 200 to 1,000 bp, as verified by gel electrophoresis. The fragmented DNA was then diluted to a concentration of 13.4 ng/μl and denatured for 10 minutes at 95°C, after which two input fractions (approximately 250 ng each), serving as references in subsequent microarray hybridizations (see below), were collected and stored at −80°C. The remaining DNA aliquots were immunoprecipitated overnight with monoclonal antibodies against either 5 mC or 5 hmC using magnetic bead-based MeDIP and hMeDIP kits (Diagenode, Liège, Belgium) to purify and isolate the precipitated DNA. Both the input fraction and the immunoprecipitated DNAs were then amplified using a WGA2 kit (Sigma-Aldrich), generating sufficient amounts (approximately 6 μg) for the various microarray hybridizations detailed below.

### Genome-wide methylation profiling

Of the methylated and input fraction DNAs, 1.5 μg was differentially labeled with Cy3 and Cy5, respectively, and hybridized to BAC array slides containing 32,433 tiling clones covering at least 98% of the human genome. The slides were produced at the SCIBLU DNA microarray resource center at Lund University, Sweden. Labeling of DNA, slide preparation, hybridization and analysis were performed as described previously [49].

Classification as relative enrichment of methylation was based on the log2 ratio for each of the BAC clones smoothed with the factor of 0.33, with a positive ratio indicating a higher degree of methylation. PCA and HCA were performed as described above. The three groups (disomy 8, trisomy 8, and references) were analyzed in relation to the gene density on each BAC clone by ascertaining, using a custom-made script, the number of annotated RefSeq genes on each consecutive clone. All BACs on each chromosome were grouped in relation to the number of genes they harbor, which most often were 0 to 3 genes per BAC (total range 0 to 20). The average log2 ratio for each group was then plotted against their gene content. For every chromosome, each gene count group, comprising at least 0.1% of the total number of BAC clones on the chromosome, was analyzed using an independent and weighted ANOVA to ascertain whether there were statistically significant differences in 5mC levels between disomy 8, trisomy 8, and references. The Tukey HSD test was applied to investigate between which types of culture the significant differences were present in. The analyses were performed using the VassarStats website (http://vassarstats.net/).

### Gene-specific methylation and hydroxymethylation profiling

A total of 4 μg of the immunoprecipitated and input fraction DNAs were analyzed using the Human DNA Methylation 2.1 M Deluxe Promoter Arrays (Roche NimbleGen, Madison, WI, USA) by the NimbleGen Service Group, who performed the quality control, labeling, hybridization, and scanning. This array platform covers all 27,867 University of California Santa Cruz (UCSC)-annotated CpG islands and promoter regions for all known RefSeq genes as well as 730 annotated miRNA promoters.

Signal intensity data were extracted from the scanned images, with each feature on the array having a corresponding log2 ratio, that is, the ratio of the input signals for the precipitated and the input fraction DNAs. PCA and HCA were performed as above. In addition, the log2 ratio was scaled in order to center the ratio data around zero by subtracting the bi-weight mean for the log2 ratio values for all features on the array from each log2 ratio value. A modified algorithm for capturing microarray enrichment (ACME) [50], where a fixed-length window (750 bp) is placed around each consecutive probe, and the one-sided Kolmogorov-Smirnov test were then applied on the scaled log2 ratio data to determine whether the probes were drawn from a significantly more positive distribution (peak) of intensity log2 ratios than the other probes in the array. The resulting score for each probe is the -log10 *P-*value from the windowed Kolmogorov-Smirnov test around that probe.

For every group (disomy 8, trisomy 8, and reference), all genes with significant CpG island/promoter methylation scores were ascertained. Then, an edge-preserving smoother analysis [51], using the Pott’s filter with the penalty parameter set to 2 and the least allowed aberration size limited to two clones, was performed to identify genes that were highly enriched for 5 mC or 5 hmC in the three different culture groups. The expression of each gene was then investigated, after which possible associations between hypermethylated and hyperhydroxymethylated promoters/CpG islands and gene expression levels were compared using a *t*-test. Underexpression and overexpression were defined as fold changes ≤ −0.5 and ≥0.5, respectively; expression levels between these cut-offs were considered ‘intermediate’.

All microarray data are minimum information about a microarray experiment (MIAME) compliant and available to download at the Gene Expression Omnibus Archive (http://www.ncbi.nlm.nih.gov/geo/) under the accession number GSE40321.

## Abbreviations

ALL: Acute lymphoblastic leukemia; bp: Base pair; CT8M: Constitutional trisomy 8 mosaicism; Chr8: Chromosome 8; DS: Down syndrome; FBS: Fetal bovine serum; FISH: Fluorescence in situ hybridization; HCA: Hierarchical cluster analysis; HSD: Honestly significant difference; MeDIP: Methylated DNA immunoprecipitation; miRNA: MicroRNA; PCA: Principal component analysis; PCR: Polymerase chain reaction; qPCR: Quantitative real-time PCR; SAM: Significance analysis of microarrays; Xi: Inactivated X chromosome.

## Competing interests

The authors declare that they have no competing interests.

## Authors’ contributions

JD and BJ participated in the concept and writing of the manuscript. JD executed specific experiments. JD, SV and BJ were involved in the analysis of the data. All authors have read and approved the final manuscript.

## Supplementary Material

Additional file 1: Table S1 Case reports on hematological disorders/malignancies in patients with CT8M. Click here for file

Additional file 2: Figure S1 Unsupervised HCA of gene and miRNA expression reveals accurate clustering of the trisomy 8 subgroup. (A) HCA of global gene expression in the disomy 8, trisomy 8, and reference cultures clustered trisomy 8 in the same branch. (B) A similar pattern was observed when co-analyzing gene and miRNA expression. (C) On the other hand, HCA of only global miRNA expression did not cluster the different subgroups accurately. (D) After long-term culturing of one trisomy 8 culture (6lt) and one disomy 8 culture (1lt), HCA of global gene and miRNA expression accurately grouped these together with their short-term cultured counterparts. Click here for file

Additional file 3: Figure S2 The majority of genes on chromosome 8 in the trisomy 8 cultures are upregulated compared with the disomy and reference cultures. Median-centered expression of genes on chromosome 8 demonstrates that the majority of these genes in the trisomy 8 cultures are upregulated compared with (A) the reference cultures as well as compared with both the disomy 8 and (B) reference cultures. Click here for file

Additional file 4: Figure S3 Validation of expression array results by real-time quantitative PCR (qPCR). Seven genes were selected from Table 1 and analyzed with qPCR using commercial Taq-Man probes. The expression levels are presented as a fold change for each culture using TBP as endogenous control. The qPCR analyses confirmed the over- or underexpression of the seven genes in the trisomy 8-positive cultures compared with the disomy 8 and reference cultures. Click here for file

Additional file 5: Table S2 Ingenuity pathway analysis of 502 differentially expressed genes in the trisomy 8 and disomy 8 cultures. Click here for file

Additional file 6: Table S3 Hypermethylated and hyperhydroxymethylated promoters/CpG islands in relation to gene expression. Click here for file

Additional file 7: Figure S4 Promoter-specific methylation levels of autosomes do not differ between the trisomy 8 and disomy 8/refererence cultures. The levels of average promoter-specific methylation on chromosomes (A) 2, (B) 6, (C) 7, and (D) 8 in the trisomy 8 cultures and in the disomy 8 and reference cultures combined were log2-converted, median-centered, and plotted against genomic positions. No significant differences between the culture groups were observed. Click here for file

Additional file 8: Figure S5 Promoter-specific hydroxymethylation levels of chromosome 8, but not of the other autosomes, is significantly lower in the trisomy 8 compared with the disomy 8/reference cultures. The levels of average promoter-specific hydroxymethylation on chromosomes (A) 2, (B) 6, (C) 7, and (D) 8 in the trisomy 8 cultures and in the disomy 8 and references cultures combined cultures were log2-converted, median-centered, and plotted against genomic positions. Significantly lower levels (*P* < 0.05; *t-*test) were observed only for chromosome 8 in the trisomy 8 cultures. Click here for file

Additional file 9: Figure S6 The X chromosome is significantly less hydroxymethylated compared with all autosomes. Measuring and comparing the average intensity ratios of all autosomes combined with the X chromosome, the promoters/CpG islands on the latter chromosome were generally (irrespective of gender and culture group) significantly (*P* <0.05; *t-*test) less hydroxymethylated. Click here for file

Additional file 10: Table S4 Candidate genes for clinical manifestations. Click here for file

Additional file 11: Figure S7 Functional association analyses of cardiovascular dysfunction, cancer, and neurological development and function reveal widespread interactions between the candidate genes. Analyses of association data including protein and genetic interactions, pathways, co-expression, co-localization, and protein domain similarity of candidate genes derived from Table 1 reveal widespread interactions between gene sets associated with (A) cardiovascular dysfunction, (B) cancer, and (C) neurological development and function. Circles in grey represent candidate genes (Table 1), whereas white circles represent associated genes identified by the GeneMANIA analyses. Since the analysis is three dimensional, the sizes of the circles vary in this two dimensional representation. The colors of the lines connecting the various genes denote the different types of functional associations, as indicated by the color boxes. Click here for file

Additional file 12: Figure S8 Schematic overview of single-cell cloning of cells disomic and trisomic for chromosome 8, respectively. From an original cell line from a patient with CT8M, with the karyotype 47,XY,+8[5]/46,XY[20], a total of three cultures with disomy 8 and three cultures with trisomy 8 were generated. DNA and RNA from these cultures were used for subsequent epigenetic analyses. In addition, two commercially available control cell lines, with normal male and female karyotypes, respectively, were included as references in all analyses. Click here for file
