# Supplementary material for: Constitutional trisomy 8 mosaicism as a model for epigenetic studies of aneuploidy
Source: Epigenetics Chromatin. 2013 Jul 1;6:18. doi: 10.1186/1756-8935-6-18 (PMC3704342; doi:10.1186/1756-8935-6-18)
Supplement: Additional file 1: Table S1 — Case reports on hematological disorders/malignancies in patients with CT8M. [file 1756-8935-6-18-S1.doc]

| **Additional file 1: Table S1** **Case reports on hematological disorders/malignancies in patients with CT8M** | | |
| --- | --- | --- |
| **Reference** | **Age/**  **sex** | **Type of**  **disorder** |
| Gafter U, Shabtal F, Kahn Y, Halbrecht I, & Djaldetti M (1976). Aplastic anemia followed by leukemia in congenital trisomy 8 mosaicism. Ultrastructural studies of polymorphonuclear cells in peripheral blood. Clin Genet 9:134-142. | 40/F | AA |
| Riccardi VM (1976). Trisomy 8 mosaicism in the skin of a patient with leukemia. Birth Defects Orig Artic Ser 12:187. | 42/M | AML |
| Riccardi VM, Humbert JR, & Peakman D (1978). Acute leukemia associated with trisomy 8 mosaicism and a familial translocation 46,XY,t(7;20)(p13;p12). Am J Med Genet 2:15-21. | 40/M | AML |
| Cornaglia-Ferraris P, et al. (1981). [Diminished in vitro colony forming capacity of bone marrow cells in a case of chromosome 8 trisomy (mosaicism): criteria for "high risk" pre-leukemia syndrome]. Boll Ist Sieroter Milan 60:69-73. | 5/M | HA |
| Palmer CG, Provisor AJ, Weaver DD, Hodes ME, & Heerema, N (1983). Juvenile chronic granulocytic leukemia in a patient with trisomy 8, neurofibromatosis, and prolonged Epstein-Barr virus infection. J Pediatr 102:888-892. | 4/M | JMML |
| Kapaun P, et al. (1993). Atypical chronic myelogenous leukemia in a patient with trisomy 8 mosaicism syndrome. Ann Hematol 66:57-58. | 17/F | CML |
| Hasle H, Clausen N, Pedersen B, & Bendix-Hansen K (1995). Myelodysplastic syndrome in a child with constitutional trisomy 8 mosaicism and normal phenotype. Cancer Genet Cytogenet 79:79-81. | 11/M | MDS |
| Mastrangelo R, Tornesello A, Mastrangelo S, Zollino M, & Neri G (1995). Constitutional trisomy 8 mosaicism evolving to primary myelodysplastic syndrome: a new subset of biologically related patients? Am J Hematol 48:67-68. | 18/M | MDS |
| Zollino M, et al. (1995). Constitutional trisomy 8 and myelodysplasia: report of a case and review of the literature. Leuk Res 19:733-736. | 18/M | MDS |

| Seghezzi L, et al. (1996). Constitutional trisomy 8 as first mutation in multistep carcinogenesis: clinical, cytogenetic, and molecular data on three cases. Genes Chromosomes Cancer 17:94-101. | 7/M  1/M  28/F | MDS-RA  ALL  MFS |
| --- | --- | --- |
| Brady AF, Waters CS, Pocha MJ, & Brueton LA (2000). Chronic myelomonocytic leukaemia in a child with constitutional partial trisomy 8 mosaicism. Clin Genet 58:142-146 | 3/M | CML |
| Maserati E, et al (2002). Trisomy 8 in myelodysplasia and acute leukemia is constitutional in 15-20% of cases. Genes Chromosomes Cancer 33:93-97. | 61/F  3/M | CML  MDS-RA |
| Narendran A, et al. (2004). Characterization of bone marrow stromal abnormalities in a patient with constitutional trisomy 8 mosaicism and myelodysplastic syndrome. Pediatr Hematol Oncol 21:209-221. | 10/F | MDS |
| Welborn J (2004). Constitutional chromosome aberrations as pathogenetic events in hematologic malignancies. Cancer Genet Cytogenet 149:137-153. | 32/M  41/F | MDS  MDS |
| Ando S, et al. (2005). Constitutional trisomy 8 mosaicism with myelodysplastic syndrome complicated by intestinal Behcet disease and antithrombin III deficiency. Cancer Genet Cytogenet 162:172-175. | 49/M | MDS |
| Maserati E, et al. (2007). Constitutional trisomy 8 mosaicism in primary myelofibrosis: relevance to clinical practice and warning for trisomy 8 studies. Cancer Genet Cytogenet 179:79-81. | 24/F | MFS |
| Yamamoto K, et al. (2007). A novel t(8;18)(q13;q21) in acute monocytic leukemia evolving from constitutional trisomy 8 mosaicism. Cancer Genet Cytogenet 176:144-149. | 38/F | AML |

Abbreviations: AA, aplastic anemia; ALL, acute lymphoblastic leukemia; AML, acute myeloid leukemia; CML, chronic myeloid leukemia; HA, hyporegenerative anemia; JMML, juvenile myelomonocytic leukemia; MDS, myelodysplastic syndrome; MDS-RA, myelodysplastic syndrome subtype refractory anemia; MFS, myelofibrosis.
