# Supplementary material for: Constitutional trisomy 8 mosaicism as a model for epigenetic studies of aneuploidy
Source: Epigenetics Chromatin. 2013 Jul 1;6:18. doi: 10.1186/1756-8935-6-18 (PMC3704342; doi:10.1186/1756-8935-6-18)
Supplement: Additional file 2: Figure S1 — Unsupervised HCA of gene and miRNA expression reveals accurate clustering of the trisomy 8 subgroup. (A) HCA of global gene expression in the disomy 8, trisomy 8, and reference cultures clustered trisomy 8 in the same branch. (B) A similar pattern was observed when co-analyzing gene and miRNA expression. (C) On the other hand, HCA of only global miRNA expression did not cluster the different subgroups accurately. (D) After long-term culturing of one trisomy 8 culture (6lt) and one disomy 8 culture (1lt), HCA of global gene and miRNA expression accurately grouped these together with their short-term cultured counterparts. [file 1756-8935-6-18-S2.doc]

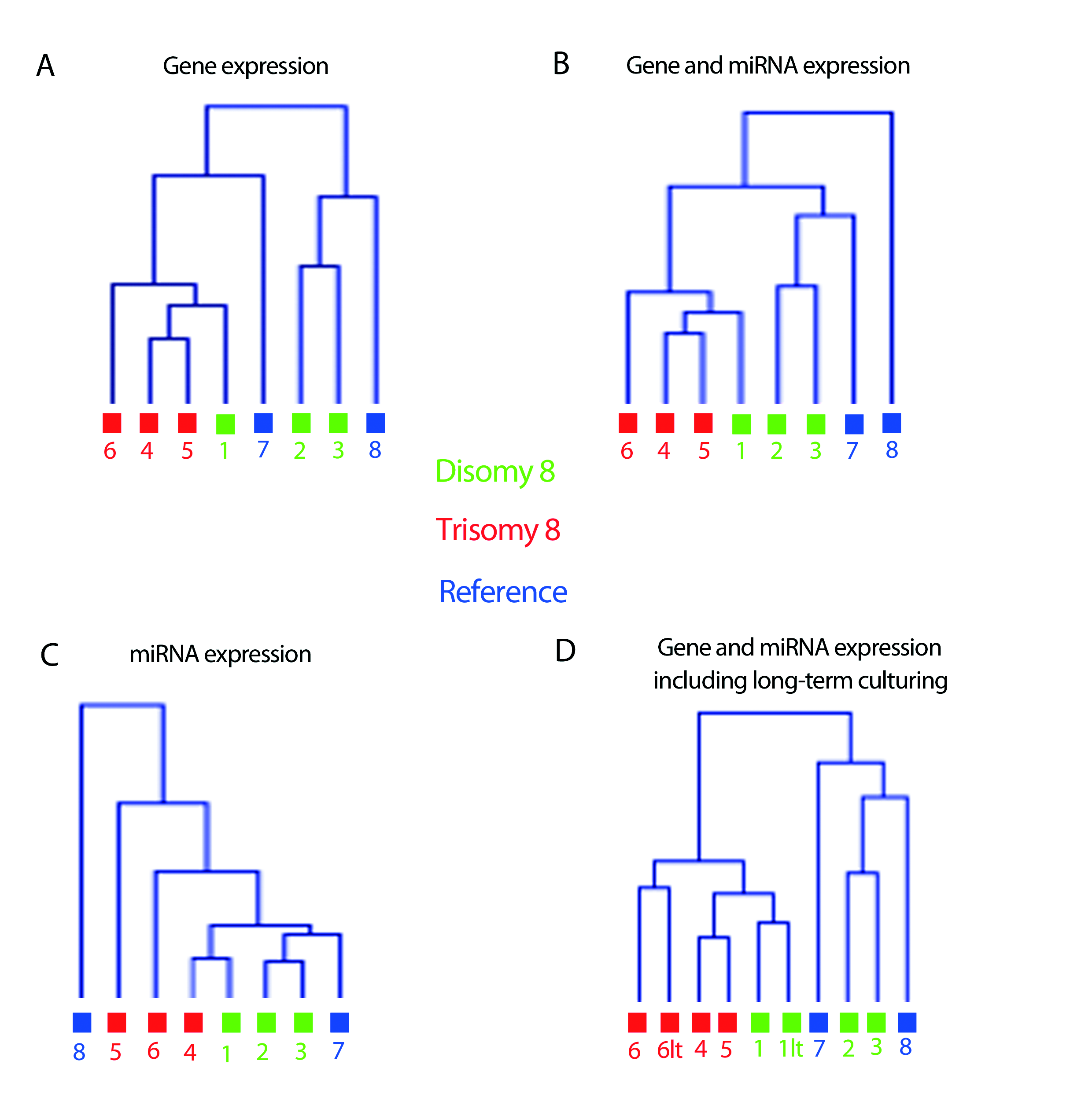


**Additional file 2: Figure S1 Unsupervised HCA of gene and miRNA expression reveals accurate clustering of the trisomy 8 subgroup.** **(A)** HCA of global gene expression in the disomy 8, trisomy 8, and reference cultures clustered trisomy 8 in the same branch. **(B)** A similar pattern was observed when co-analyzing gene and miRNA expression. **(C)** On the other hand, HCA of only global miRNA expression did not cluster the different subgroups accurately. **(D)** After long-term culturing of one trisomy 8 culture (6lt) and one disomy 8 culture (1lt), HCA of global gene and miRNA expression accurately grouped these together with their short-term cultured counterparts.
