# Supplementary material for: Constitutional trisomy 8 mosaicism as a model for epigenetic studies of aneuploidy
Source: Epigenetics Chromatin. 2013 Jul 1;6:18. doi: 10.1186/1756-8935-6-18 (PMC3704342; doi:10.1186/1756-8935-6-18)
Supplement: Additional file 3: Figure S2 — The majority of genes on chromosome 8 in the trisomy 8 cultures are upregulated compared with the disomy and reference cultures. Median-centered expression of genes on chromosome 8 demonstrates that the majority of these genes in the trisomy 8 cultures are upregulated compared with (A) the reference cultures as well as compared with both the disomy 8 and (B) reference cultures. [file 1756-8935-6-18-S3.doc]

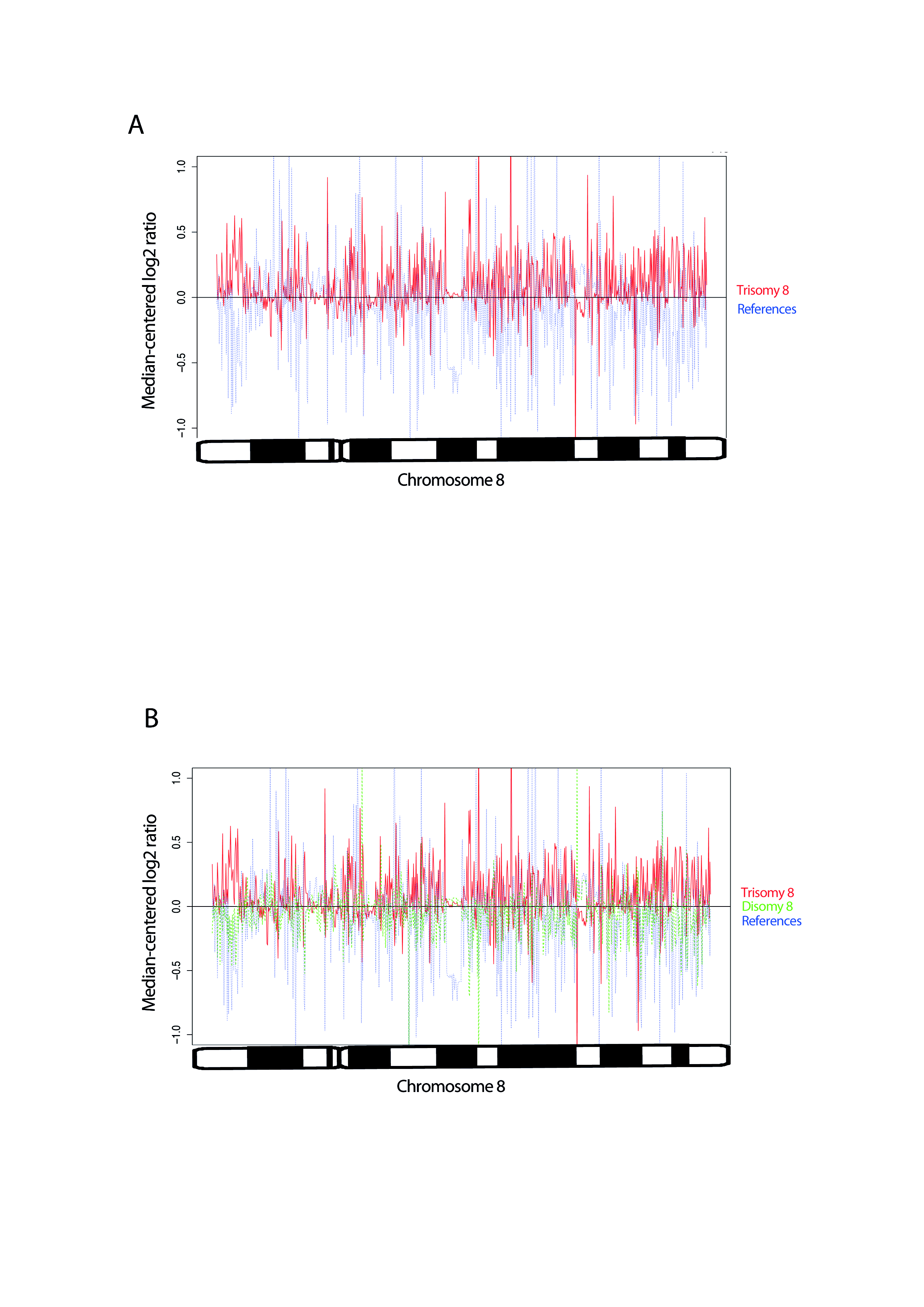


**Additional file 3: Figure S2 The majority of genes on chromosome 8 in the trisomy 8 cultures are upregulated compared with the disomy and reference cultures.** Median-centered expression of genes on chromosome 8 demonstrates that the majority of these genes in the trisomy 8 cultures are upregulated compared with **(A)** the reference cultures as well as compared with both the disomy 8 and **(B)** reference cultures.
