# Supplementary material for: Constitutional trisomy 8 mosaicism as a model for epigenetic studies of aneuploidy
Source: Epigenetics Chromatin. 2013 Jul 1;6:18. doi: 10.1186/1756-8935-6-18 (PMC3704342; doi:10.1186/1756-8935-6-18)
Supplement: Additional file 4: Figure S3 — Validation of expression array results by real-time quantitative PCR (qPCR). Seven genes were selected from Table 1 and analyzed with qPCR using commercial Taq-Man probes. The expression levels are presented as a fold change for each culture using TBP as endogenous control. The qPCR analyses confirmed the over- or underexpression of the seven genes in the trisomy 8-positive cultures compared with the disomy 8 and reference cultures. [file 1756-8935-6-18-S4.docx]

**Additional file 4: Figure S3 Validation of expression array results by real-time quantitative PCR (qPCR).** Seven genes were selected from Table 1 and analyzed with qPCR using commercial Taq-Man probes. The expression levels are presented as a fold change for each cultures using *TBP* as endogenous control. The qPCR analyses confirmed the over- or underexpression of the seven genes in the trisomy 8-positive cultures compared with the disomy 8 and reference cultures.
