# Supplementary material for: Constitutional trisomy 8 mosaicism as a model for epigenetic studies of aneuploidy
Source: Epigenetics Chromatin. 2013 Jul 1;6:18. doi: 10.1186/1756-8935-6-18 (PMC3704342; doi:10.1186/1756-8935-6-18)
Supplement: Additional file 5: Table S2 — Ingenuity pathway analysis of 502 differentially expressed genes in the trisomy 8 and disomy 8 cultures. [file 1756-8935-6-18-S5.doc]

| **Additional file 5: Table S2 Ingenuity pathway analysis of 502 differentially expressed genes in the trisomy 8 and disomy 8 cultures*^a^*** | | |
| --- | --- | --- |
| **Diseases and disorders** | ***P* value** | **No. of Genes** |
| Cancer | <0.05 | 108 |
| Genetic disorder | <0.05 | 69 |
| Hypersensitivity response | <0.01 | 2 |
| Inflammatory response | <0.01 | 2 |
| Skeletal and muscular disorders | <0.05 | 13 |
| **Molecular and cellular function** | ***P* value** | **No. of Genes** |
| Protein synthesis | <0.01 | 20 |
| Cell cycle | <0.05 | 11 |
| Cell morphology | <0.05 | 17 |
| Cellular development | <0.05 | 36 |
| Cell death | <0.05 | 17 |
| **Physiological system development and function** | ***P* value** | **No. of Genes** |
| Hematological system development and function | <0.05 | 6 |
| Hematopoiesis | <0.05 | 6 |
| Tissue morphology | <0.05 | 10 |
| Tissue development | <0.05 | 5 |

*^a^*Ascertained by a *t*-test of the global gene expression patterns in the trisomy 8 and

disomy 8 cultures.
