# Supplementary material for: Constitutional trisomy 8 mosaicism as a model for epigenetic studies of aneuploidy
Source: Epigenetics Chromatin. 2013 Jul 1;6:18. doi: 10.1186/1756-8935-6-18 (PMC3704342; doi:10.1186/1756-8935-6-18)
Supplement: Additional file 6: Table S3 — Hypermethylated and hyperhydroxymethylated promoters/CpG islands in relation to gene expression. [file 1756-8935-6-18-S6.doc]

| **Additional file 6: Table S3 Hypermethylated and hyperhydroxymethylated promoters/CpG islands in relation to gene expression** | | | |
| --- | --- | --- | --- |
| **Cultures** | **Groups** | **No. of hypermethylated genes**  **(% under- /over-/intermediately expressed)** | **No. of hyperhydroxymethylated genes**  **(% under- /over-/intermediately expressed)** |
| 1,2,3 | Disomy 8 | 158 (77/2/21) | 198 (20/26/54) |
| 4,5,6 | Trisomy 8 | 161 (83/3/14) | 203 (27/33/40) |
| 7,8 | Reference | 144 (84/1/15) | 187 (25/26/49) |
