# Supplementary material for: Constitutional trisomy 8 mosaicism as a model for epigenetic studies of aneuploidy
Source: Epigenetics Chromatin. 2013 Jul 1;6:18. doi: 10.1186/1756-8935-6-18 (PMC3704342; doi:10.1186/1756-8935-6-18)
Supplement: Additional file 7: Figure S4 — Promoter-specific methylation levels of autosomes do not differ between the trisomy 8 and disomy 8/refererence cultures. The levels of average promoter-specific methylation on chromosomes (A) 2, (B) 6, (C) 7, and (D) 8 in the trisomy 8 cultures and in the disomy 8 and reference cultures combined were log2-converted, median-centered, and plotted against genomic positions. No significant differences between the culture groups were observed. [file 1756-8935-6-18-S7.doc]

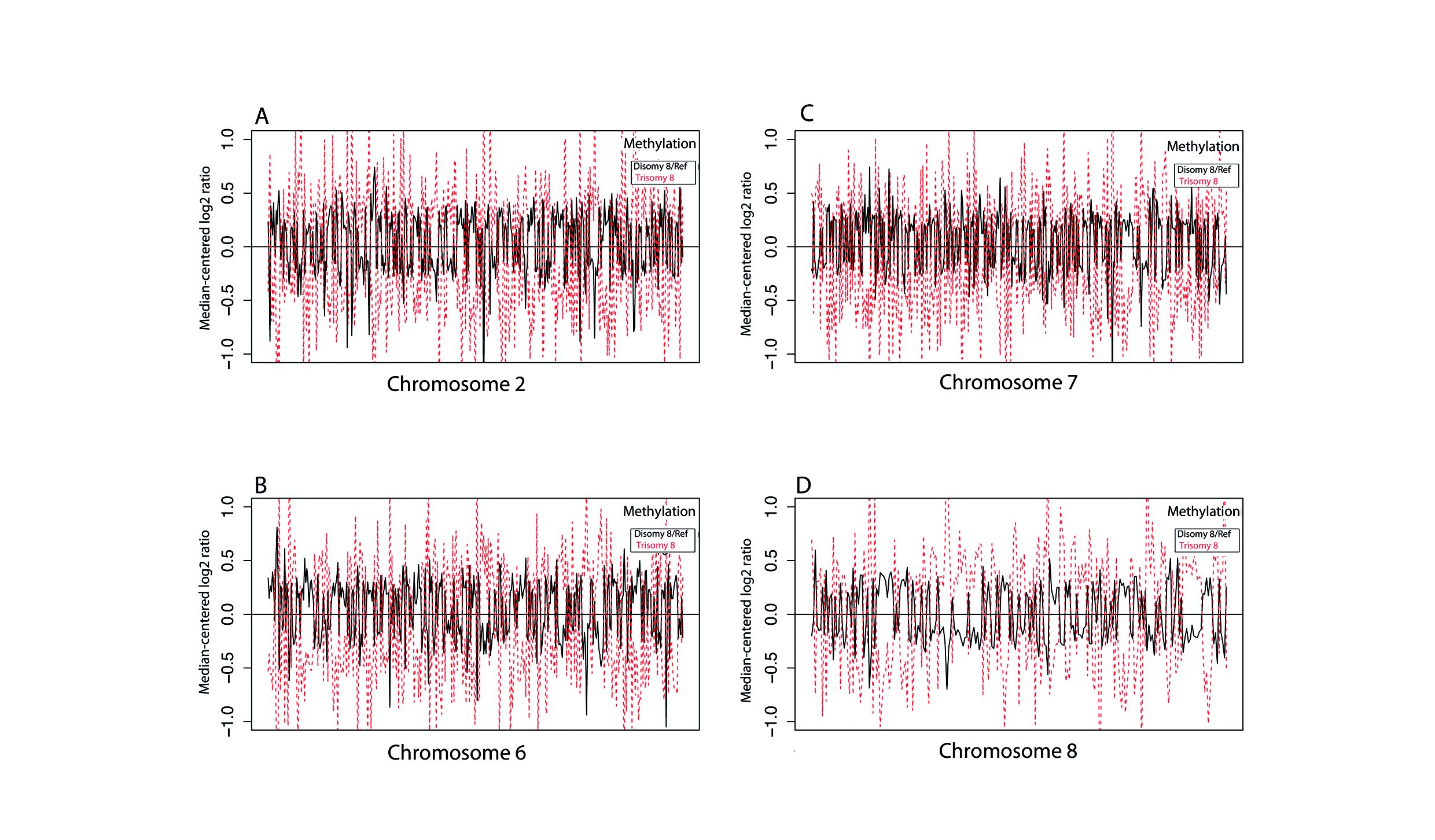


**Additional file 7: Figure S4 Promoter-specific methylation levels of autosomes do not differ between the trisomy 8 and disomy 8/refererence cultures.** The levels of average promoter-specific methylation on chromosomes **(A)** 2, **(B)** 6, **(C)** 7, and **(D)** 8 in the trisomy 8 cultures and in the disomy 8 and reference cultures combined were log2-converted, median-centered, and plotted against genomic positions. No significant differences between the culture groups were observed.
