# Supplementary material for: Constitutional trisomy 8 mosaicism as a model for epigenetic studies of aneuploidy
Source: Epigenetics Chromatin. 2013 Jul 1;6:18. doi: 10.1186/1756-8935-6-18 (PMC3704342; doi:10.1186/1756-8935-6-18)
Supplement: Additional file 8: Figure S5 — Promoter-specific hydroxymethylation levels of chromosome 8, but not of the other autosomes, is significantly lower in the trisomy 8 compared with the disomy 8/reference cultures. The levels of average promoter-specific hydroxymethylation on chromosomes (A) 2, (B) 6, (C) 7, and (D) 8 in the trisomy 8 cultures and in the disomy 8 and references cultures combined cultures were log2-converted, median-centered, and plotted against genomic positions. Significantly lower levels (P < 0.05; t-test) were observed only for chromosome 8 in the trisomy 8 cultures. [file 1756-8935-6-18-S8.doc]

**
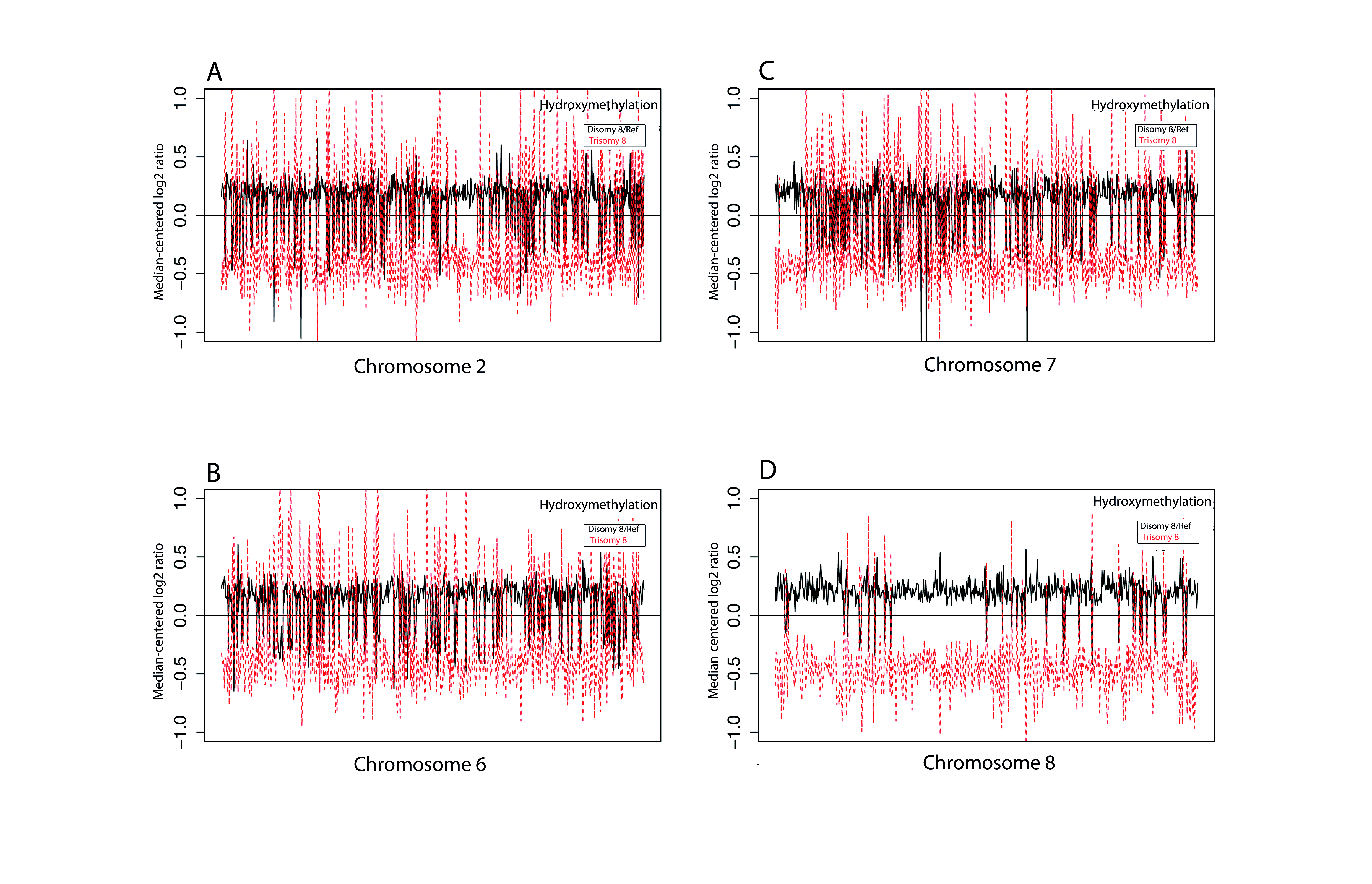
**

**Additional file 8: Figure S5 Promoter-specific hydroxymethylation levels of chromosome 8, but not of the other autosomes, is significantly lower in the trisomy 8 compared with the disomy 8/reference cultures.** The levels of average promoter-specific hydroxymethylation on chromosomes **(A)** 2, **(B)** 6, **(C)** 7, and **(D)** 8 in the trisomy 8 cultures and in the disomy 8 and references cultures combined cultures were log2-converted, median-centered, and plotted against genomic positions. Significantly lower levels (*P* < 0.05; *t-*test) were observed only for chromosome 8 in the trisomy 8 cultures.
