# Supplementary material for: Constitutional trisomy 8 mosaicism as a model for epigenetic studies of aneuploidy
Source: Epigenetics Chromatin. 2013 Jul 1;6:18. doi: 10.1186/1756-8935-6-18 (PMC3704342; doi:10.1186/1756-8935-6-18)
Supplement: Additional file 9: Figure S6 — The X chromosome is significantly less hydroxymethylated compared with all autosomes. Measuring and comparing the average intensity ratios of all autosomes combined with the X chromosome, the promoters/CpG islands on the latter chromosome were generally (irrespective of gender and culture group) significantly (P <0.05; t-test) less hydroxymethylated. [file 1756-8935-6-18-S9.doc]

**
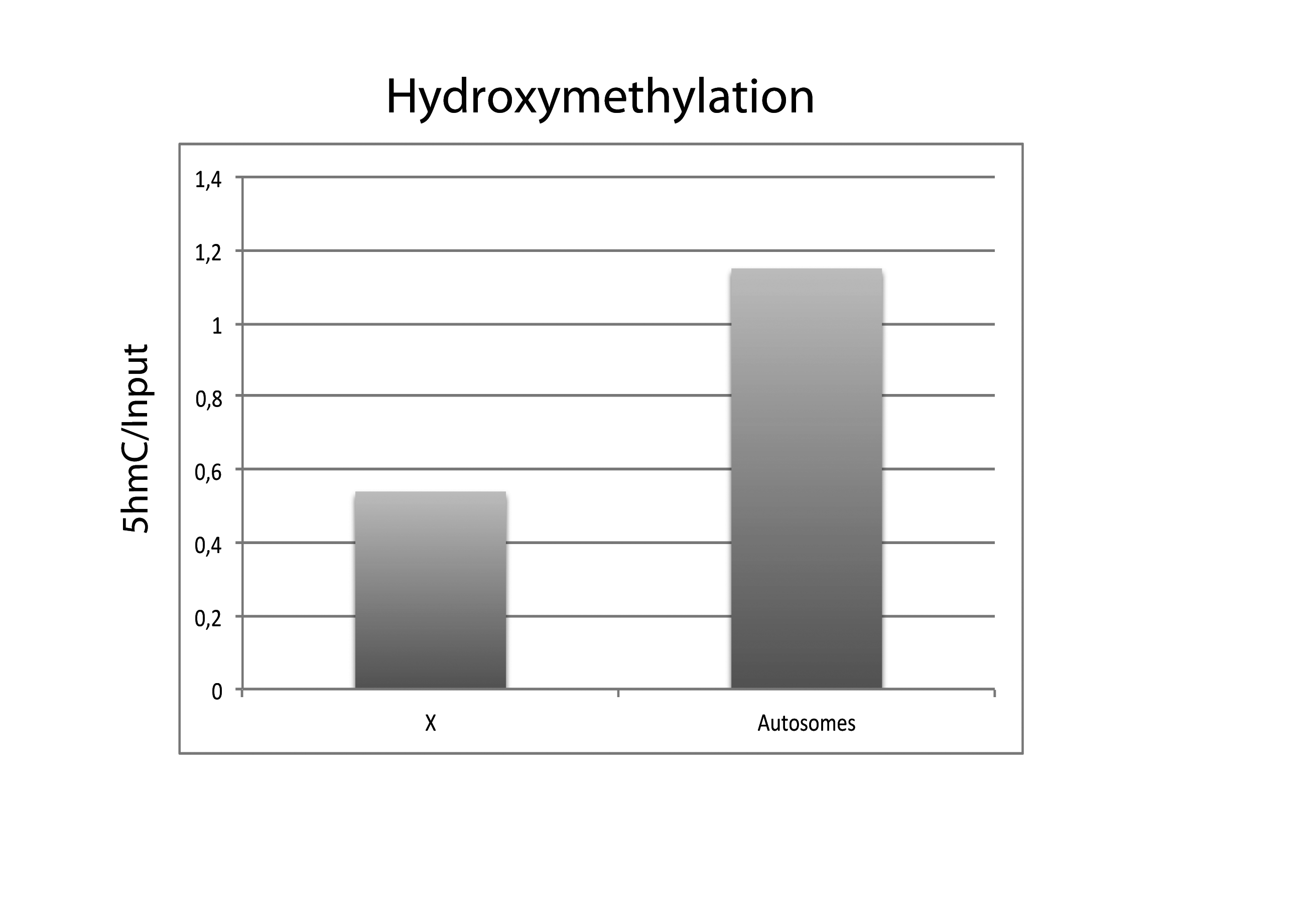
**

**Additional file 9: Figure S6 The X chromosome is significantly less hydroxymethylated compared with all autosomes.** Measuring and comparing the average intensity ratios of all autosomes combined with the X chromosome, the promoters/CpG islands on the latter chromosome were generally (irrespective of gender and culture group) significantly (*P* < 0.05; *t-*test) less hydroxymethylated.
