# Supplementary material for: Constitutional trisomy 8 mosaicism as a model for epigenetic studies of aneuploidy
Source: Epigenetics Chromatin. 2013 Jul 1;6:18. doi: 10.1186/1756-8935-6-18 (PMC3704342; doi:10.1186/1756-8935-6-18)
Supplement: Additional file 10: Table S4 — Candidate genes for clinical manifestations. [file 1756-8935-6-18-S10.doc]

**Additional file 10: Table S4 Candidate genes for clinical manifestations**

| **Gene** | **Mani-**  **festation** | **Reference** |
| --- | --- | --- |
| *AGPAT6* | MR | Li D, et al. (2003) Cloning and identification of the human LPAAT-zeta gene, a novel member of the lysophosphatidic acid acyltransferase family. J Hum Genet 48:438-442. |
| *CASP4* | ST | Mao ZG, et al. (2010) TRAIL-induced apoptosis of human melanoma cells involves activation of caspase-4. Apoptosis 15:1211-1222. |
| *COX6C* | ST | Wang FL, et al. (1996) Two differentially expressed genes in normal human prostate tissue and in carcinoma. Cancer Res 56:3634-3637. |
| *CPXM2* | MR | Xin X, Day R, Dong W, Lei Y, & Fricker LD (1998) Identification of mouse CPX-2, a novel member of the metallocarboxypeptidase gene family: cDNA cloning, mRNA distribution, and protein expression and characterization. DNA Cell Biol 17:897-909. |
| *CRYAB* | MR | Sacconi S, et al. (2012) A novel CRYAB mutation resulting in multisystemic disease. Neuromuscul Disord 22:66-72. |
| *DES* | CM | van Spaendonck-Zwarts K, et al. (2011) Desmin-related myopathy: a review and meta-analysis. Clin Genet 80:354-366. |
| *EDNRA* | MR | Joshi G, Pradhan S, & Mittal B (2011) Vascular gene polymorphisms (EDNRA -231 G>A and APOE HhaI) and risk for migraine. DNA Cell Biol 30:577-584. |
| *ELP3* | MR | Kim JH, Lane WS, & Reinberg D (2002) Human Elongator facilitates RNA polymerase II transcription through chromatin. Proc Natl Acad Sci U S A 99:1241-1246.  Creppe C, et al. (2009) Elongator controls the migration and differentiation of cortical neurons through acetylation of alpha-tubulin. Cell 136:551-564. |
| *ENTPD4* | MR | Biederbick A, Kosan C, Kunz J, & Elsässer HP (2000) First apyrase splice variants have different enzymatic properties. J Biol Chem 275:19018-19024. |

| *FNBP1* | ST | Yamamoto H, et al. (2011) Requirement for FBP17 in invadopodia formation by invasive bladder tumor cells. J Urol 185:1930-1938. |
| --- | --- | --- |
| *LDB3* | CM | Rampersaud E, et al. (2010) Common susceptibility variants examined for association with dilated cardiomyopathy. Ann Hum Genet 74:110-116. |
| *LRP12* | ST | Garnis C, Coe BP, Zhang L, Rosin MP, & Lam WL (2004) Overexpression of LRP12, a gene contained within an 8q22 amplicon identified by high-resolution array CGH analysis of oral squamous cell carcinomas. Oncogene 23:2582-2586. |
| *MIR151* | ST | Ding J, et al. (2010) Gain of miR-151 on chromosome 8q24.3 facilitates tumour cell migration and spreading through downregulating RhoGDIA. Nat Cell Biol 12:390-399.  Barnabas N, Xu L, Savera A, Hou Z, & Barrack ER (2011) Chromosome 8 markers of metastatic prostate cancer in African American men: gain of the MIR151 gene and loss of the NKX3-1 gene. Prostate 71:857-871. |
| *POPDC2* | CM | Andrée B, et al. (2000) Isolation and characterization of the novel popeye gene family expressed in skeletal muscle and heart. Dev Biol 223:371-382. |
| *RAB2A* | ST  MR | Lomnytska MI, et al. (2010) Diagnostic protein marker patterns in squamous cervical cancer. Proteomics Clin Appl 4:17-31.  Short B, et al. (2001) A GRASP55-rab2 effector complex linking Golgi structure to membrane traffic. J Cell Biol 155:877-883. |
| *RAB3GAP2* | MR | Bem D, et al. (2011) Loss-of-function mutations in RAB18 cause Warburg micro syndrome. Am J Hum Genet 88:499-507. |
| *RPS6KA1* | ST | Lara R, et al. (2011) An siRNA screen identifies RSK1 as a key modulator of lung cancer metastasis. Oncogene 30:3513-3521. |
| *SLC4A3* | CM | Chen HP, et al. (2011) Anion exchanger 3 is required for sasanquasaponin to inhibit ischemia/reperfusion-induced elevation of intracellular Cl- concentration and to elicit cardioprotection. J Cell Biochem 112:2803-2812. |

| *SNORD115-32* | MR | Runte M, et al. (2001) The IC-SNURF-SNRPN transcript serves as a host for multiple small nucleolar RNA species and as an antisense RNA for UBE3A. Hum Mol Genet 10:2687-2700.  Kishore S & Stamm S (2006) The snoRNA HBII-52 regulates alternative splicing of the serotonin receptor 2C. Science 311:230-232. |
| --- | --- | --- |
| *TNFRSF10A* | ST | Razumilava N, et al. (2012) miR-25 targets TNF-related apoptosis inducing ligand (TRAIL) death receptor-4 and promotes apoptosis resistance in cholangiocarcinoma. Hepatology 55:465-475. |
| *TNKS* | ST | Gao J, Zhang J, Long Y, Tian Y, & Lu X (2011) Expression of tankyrase 1 in gastric cancer and its correlation with telomerase activity. Pathol Oncol Res 17:685-690. |

Abbreviations: CM, cardiomyopathy; MR, mental retardation; ST, solid tumor.
