# Supplementary material for: Constitutional trisomy 8 mosaicism as a model for epigenetic studies of aneuploidy
Source: Epigenetics Chromatin. 2013 Jul 1;6:18. doi: 10.1186/1756-8935-6-18 (PMC3704342; doi:10.1186/1756-8935-6-18)
Supplement: Additional file 11: Figure S7 — Functional association analyses of cardiovascular dysfunction, cancer, and neurological development and function reveal widespread interactions between the candidate genes. Analyses of association data including protein and genetic interactions, pathways, co-expression, co-localization, and protein domain similarity of candidate genes derived from Table 1 reveal widespread interactions between gene sets associated with (A) cardiovascular dysfunction, (B) cancer, and (C) neurological development and function. Circles in grey represent candidate genes (Table 1), whereas white circles represent associated genes identified by the GeneMANIA analyses. Since the analysis is three dimensional, the sizes of the circles vary in this two dimensional representation. The colors of the lines connecting the various genes denote the different types of functional associations, as indicated by the color boxes. [file 1756-8935-6-18-S11.doc]

**
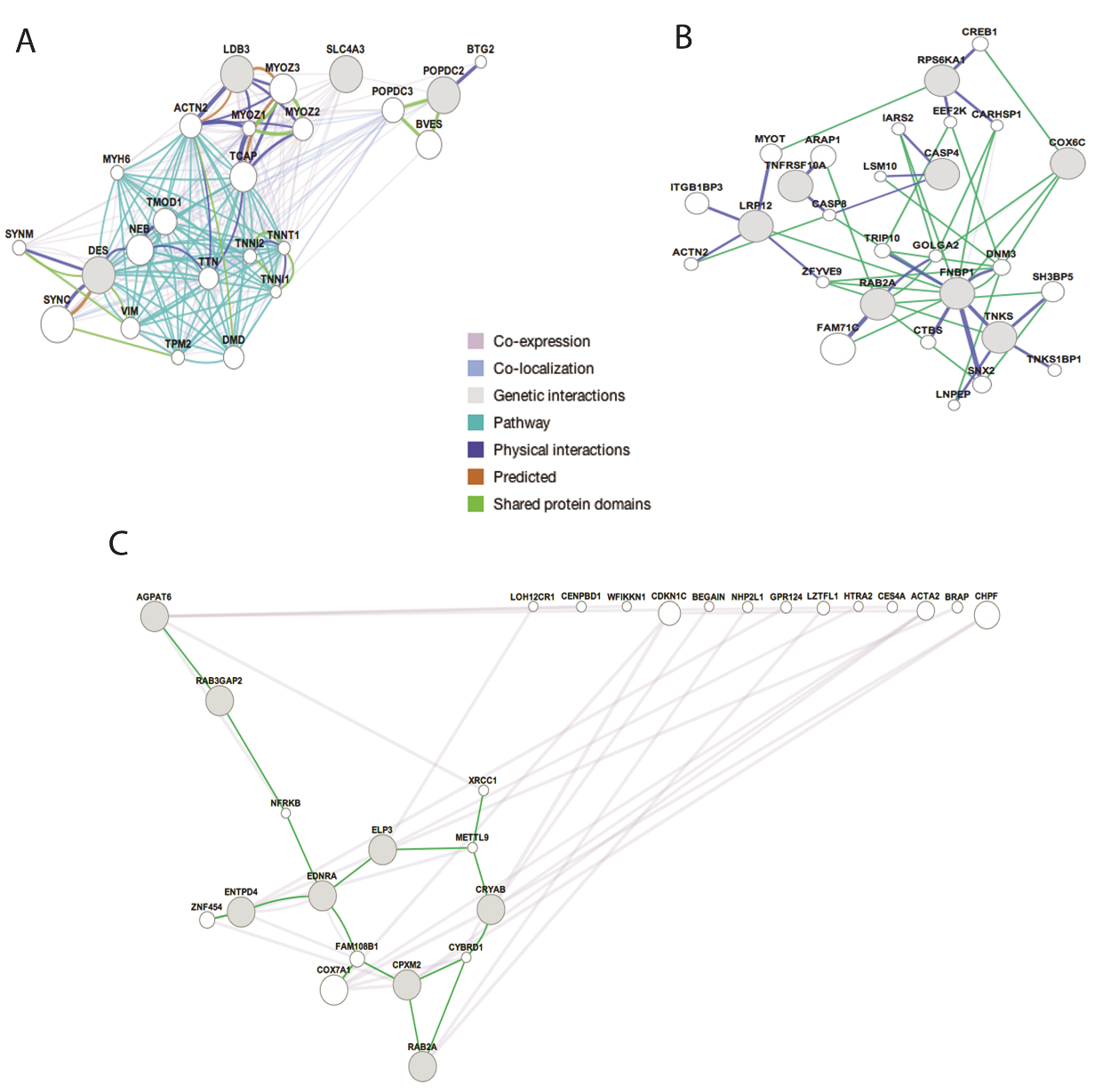
**

**Additional file 11: Figure S7 Functional association analyses of cardiovascular dysfunction, cancer, and neurological development and function reveal widespread interactions between the candidate genes.** Analyses of association data including protein and genetic interactions, pathways, co-expression, co-localization, and protein domain similarity of candidate genes derived from Table 1 reveal widespread interactions between gene sets associated with **(A)** cardiovascular dysfunction, **(B)** cancer, and **(C)** neurological development and function. Circles in grey represent candidate genes (Table 1), whereas white circles represent associated genes identified by the GeneMANIA analyses. Since the analysis is three dimensional, the sizes of the circles vary in this two dimensional representation. The colors of the lines connecting the various genes denote the different types of functional associations, as indicated by the color boxes.
