# Supplementary material for: Constitutional trisomy 8 mosaicism as a model for epigenetic studies of aneuploidy
Source: Epigenetics Chromatin. 2013 Jul 1;6:18. doi: 10.1186/1756-8935-6-18 (PMC3704342; doi:10.1186/1756-8935-6-18)
Supplement: Additional file 12: Figure S8 — Schematic overview of single-cell cloning of cells disomic and trisomic for chromosome 8, respectively. From an original cell line from a patient with CT8M, with the karyotype 47,XY,+8[5]/46,XY[20], a total of three cultures with disomy 8 and three cultures with trisomy 8 were generated. DNA and RNA from these cultures were used for subsequent epigenetic analyses. In addition, two commercially available control cell lines, with normal male and female karyotypes, respectively, were included as references in all analyses. [file 1756-8935-6-18-S12.doc]

**
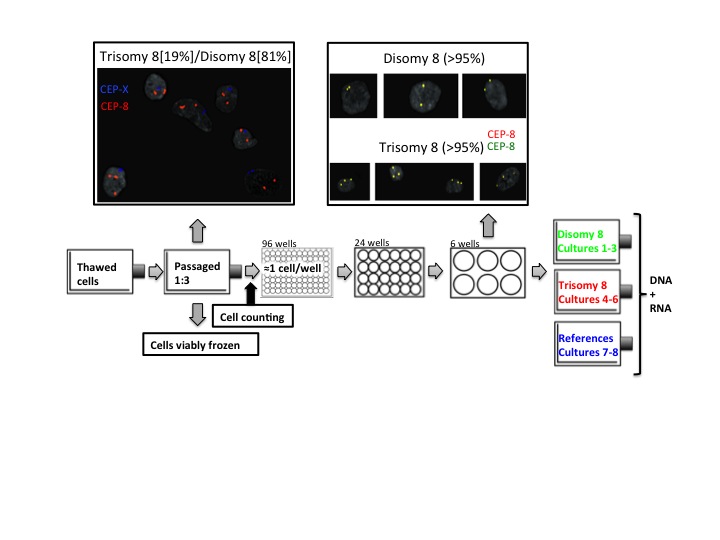
**

**Additional file 12: Figure S8** **Schematic overview of single-cell cloning of cells disomic and trisomic for chromosome 8, respectively.** From an original cell line from a patient with CT8M, with the karyotype 47,XY,+8[5]/46,XY[20], a total of three cultures with disomy 8 (green) and three cultures with trisomy 8 (red) were generated. DNA and RNA from these cultures were used for subsequent epigenetic analyses. In addition, two commercially available control cell lines (blue), with normal male and female karyotypes, respectively, were included as references in all analyses.
